# Executive functions mediate the association between ADHD symptoms and anxiety in a clinical adolescent population

**DOI:** 10.3389/fpsyt.2022.834356

**Published:** 2022-09-12

**Authors:** Anne-Lise Juul Haugan, Anne Mari Sund, Per Hove Thomsen, Stian Lydersen, Torunn Stene Nøvik

**Affiliations:** ^1^Department of Mental Health, Regional Centre for Child and Youth Mental Health and Child Welfare (RKBU), Faculty of Medicine and Health Sciences, Norwegian University of Science and Technology (NTNU), Trondheim, Norway; ^2^Department of Child and Adolescent Psychiatry, St. Olav University Hospital, Trondheim, Norway; ^3^Department of Child and Adolescent Psychiatry, Aarhus University Hospital, Aarhus, Denmark

**Keywords:** attention-deficit/hyperactivity disorder, executive functions, functional impairment, adolescents, anxiety

## Abstract

**Objective:**

Attention-deficit/hyperactivity disorder (ADHD) is associated with a high prevalence of anxiety disorders in children and adolescents. The reasons for this association are poorly understood. Preliminary findings with young adults have suggested that executive functions and functional impairment may mediate the relationship between symptoms of ADHD and mixed anxiety and depressive symptoms. The objective of this study was to explore whether ADHD symptoms, executive functions and functional impairment predict anxiety in a clinical adolescent population. In addition, we investigated the possible mediating role of executive functions and functional impairment in this relationship.

**Method:**

One hundred adolescents with ADHD and their parents completed the ADHD Rating Scale IV (ADHD RS-IV), the Behavior Rating Inventory of Executive Function (BRIEF), and the Weiss Functional Impairment Rating Scale (WFIRS) in relation to an RCT study. The adolescents also completed the Screen for Child Anxiety-Related Emotional Disorders (SCARED). Analyses were conducted using regression and a serial multiple mediator model.

**Results:**

In the regression analyses, parent-rated ADHD symptoms were unable to predict anxiety, but ADHD inattention symptoms predicted anxiety in the self-ratings. Executive dysfunction and functional impairment predicted anxiety in both the parent- and self-reports. In the mediation analyses ADHD symptoms alone did not predict anxiety, but executive dysfunction mediated this relationship as expected. Functional impairment mediated this relationship indirectly through executive functions. The results were similar in the parent- and self- reports.

**Conclusion:**

The results pinpoint executive dysfunction as an important treatment target for alleviating anxiety in adolescents with impairing ADHD symptoms.

## Introduction

Attention-deficit/hyperactivity disorder (ADHD) is one of the most common neurodevelopmental disorders in childhood (1) and is characterized by developmentally inappropriate levels of inattention, hyperactivity and impulsivity that lead to impairment in at least one life domain (2). Through adolescence, symptoms of ADHD typically impair functioning in various contexts, such as school, social settings, and emotional wellbeing. Comorbid psychiatric conditions are common in this patient group, and anxiety, depression, conduct disorders and substance misuse are among the most common disorders (3–5). Although pharmacological treatment for ADHD has shown beneficial effects on core ADHD symptoms as well as improvements in functional impairment and health-related quality of life (6, 7), the lack of data on longer-term treatment effects makes it unclear whether the changes in health-related quality of life are mediated by symptom changes, changes in functional impairment or other factors.

Anxiety disorders (ADs) are among the most prevalent disorders in child and adolescent populations, and ADHD and AD are comorbid with each other in 25–50% of cases (8–10). This comorbidity rate is greater than chance and is still present after controlling for overlapping symptoms, such as difficulty concentrating and restlessness (11, 12). The presence of both disorders is associated with more attentional problems, school fears and lower social competence than the presence of either ADHD or anxiety alone (8). While there is evidence of a prospective relationship between ADHD and AD across development, the reasons for this association are still unclear. Previous studies have suggested a specific relationship between the ADHD inattentive type and anxiety (9, 13, 14). Similarly, ADHD patients with sluggish (slow) cognitive speed have shown more internalizing symptoms, such as anxiety and depression, than patients with the hyperactive or combined subtype of ADHD (15, 16). Jensen et al. (17) found that anxiety reported in ADHD populations may differ from anxiety in non-ADHD populations, with concerns about competency and performance being the more common components rather than specific phobias *per se*. This form of anxiety has been suggested to arise primarily when one's cognitive processing abilities are overwhelmed by the demands of the environment (18). Following from this, two possible factors linking ADHD and anxiety are executive functions (EFs) and functional impairment. EFs represent higher-order cognitive processes that help us achieve our daily goals (19–21). EFs are mainly supported by the prefrontal cortex and typically include planning skills, response inhibition, mental flexibility, working memory, initiation and set shifting (22). Dysfunctional EFs may prevent the acquisition and implementation of skills, leading to difficulties handling everyday challenges related to academic functioning (23), interpersonal problems (24), and mental health (25–27). Although dysfunctional EFs are common in ADHD and have been hypothesized to underlie the functional impairments related to this disorder (28, 29), these cognitive dysfunctions are not restricted to ADHD but are rather common in various psychiatric disorders, including mood disorders and ADs (30, 31).

Previous research with university students with ADHD has demonstrated that executive functions may predict functional impairments. Dvorsky and Langberg (32) showed that executive functions, including motivation and emotional regulation skills mediated the association between ADHD symptoms and overall daily functioning. They also found organizational skills to mediate the association between ADHD symptoms and academic achievement rated by grade point average. Dorr and Armstrong (33) found that high executive functions were related to lower levels of functional impairment in patients with low ADHD symptoms, but high EF was not associated with low functional impairment in a sample of university students with a high level of ADHD symptoms. Research conducted with adolescents with ADHD has demonstrated that dysfunctional EFs are related to multiple domains of impairment even after controlling for symptoms of ADHD (23, 34). In particular, the metacognitive aspects of EFs (e.g., mental flexibility, planning and organization) have proven salient for school functioning and homework completion in this age group (34, 35). As children and adolescents with ADHD experience functional impairment in multiple domains, including school, the social arena and family life, they are also more vulnerable to developing low self-esteem. In addition, they are more often involved in risky behavior than their non-ADHD peers (36). Since functional impairment and the accompanying feeling of incompetence may trigger stress and anxiousness, it is important to explore the role of functional impairment in the ADHD- anxiety relationship, as this may guide our understanding of underlying mechanisms for this association and help us develop more targeted treatment interventions for this patient group.

To our knowledge, only one published study has explored the relationship between symptoms of ADHD and mixed anxiety and depressive symptoms with a particular emphasis on EFs and functional impairment. In this study, EFs and functional problems explained 42 to 53% of the variance in mixed anxiety and depressive symptoms in a population of university students (37). A limitation of this study was the lack of a systematic diagnostic assessment in the population.

**Figure 1:**
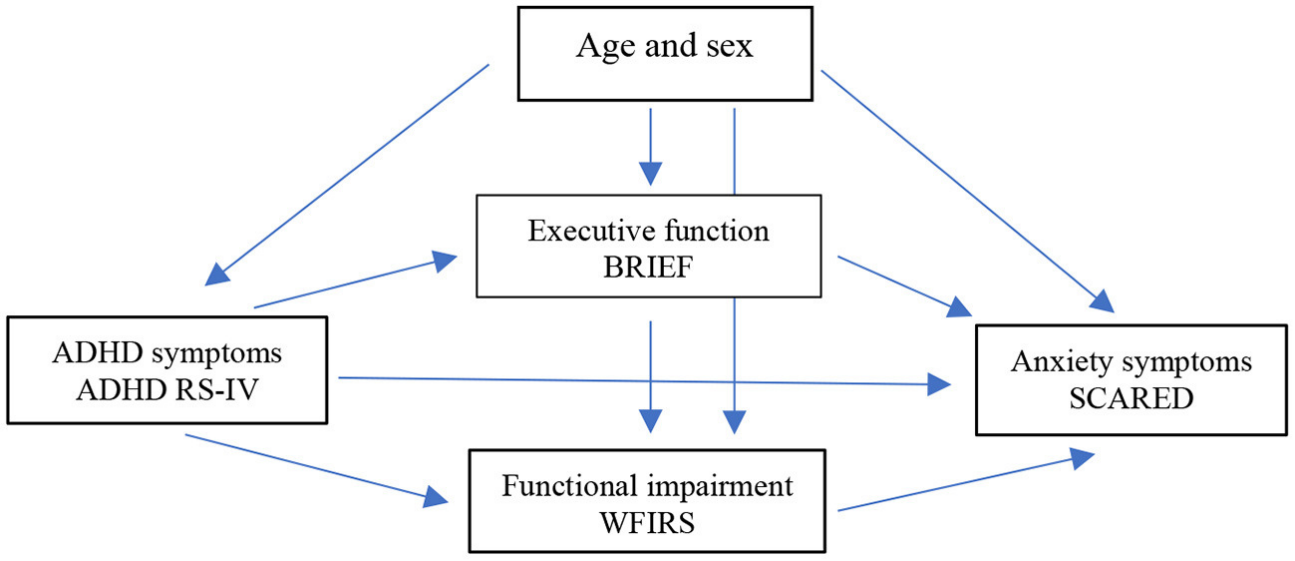
Hypothetic relationships among ADHD symptoms, functional impairment, executive dysfunction, and anxiety symptoms with the accompanying measurements. Age and sex are potential confounders.

The aim of the present study was thus to explore whether ADHD symptoms, executive dysfunction and functional impairment predict anxiety in a clinical adolescent population. Specifically, we hypothesize that the effect of ADHD symptoms on anxiety is to some extent mediated through EF or functional impairment (see Figure 1). Based on previous results (37), we expected both EFs and functional impairment to have a mediating effect on the ADHD-anxiety relationship.

## Method

### Study design and procedure

This observational study was conducted in Mid-Norway. The data were derived from a randomized controlled trial (RCT) examining the efficacy of cognitive behavioral group therapy as a follow-up treatment in a sample of adolescents with ADHD who previously received a short psychoeducational intervention and were medicated but still presented impairing ADHD symptoms (38). We refer readers to this published study and the study protocol for more details about the procedures (38, 39). Recruitment and data collection began in February 2017, and the last data were collected in September 2019. Written informed consent was obtained from the adolescents and from parents for participants under 16 years of age. The questionnaires were completed under the surveillance of a research assistant. Parent-rated questionnaires were completed by the primary caregiver, usually the mother. The study was approved by the Regional Committee for Medical and Health Research Ethics in Southeast Norway (2015/2115). The present study uses baseline data from the RCT.

### Participants

See Table 1 for the participant characteristics. The participants were between the ages of 14 and 18 years and were recruited from two outpatient child and adolescent psychiatry (CAP) units at St. Olavs Hospital. The participants had received a prior diagnosis of hyperkinetic disorder by a certified psychologist or a psychiatrist according to the International Statistical Classification of Disease and Related Health Problems (ICD-10) criteria (40). The assessment and diagnosis of hyperkinetic disorder requires a thorough clinical assessment, including a developmental history, a somatic assessment, and an examination of comorbid psychiatric disorders, and the use of questionnaires to assess ADHD symptoms (ADHD rating scale), which were completed by adolescents, parents, and teachers. The diagnostic criteria for hyperkinetic disorder in ICD-10 resemble the criteria for ADHD combined presentation in the Diagnostic and Statistical Manual of Mental Disorder 5^th^ edition (DSM-5). According to Norwegian ADHD guidelines (41), hyperkinetic disorder may also be diagnosed in patients with severe inattention symptoms, similar to the DSM-5 ADHD inattentive type. At study enrolment, each participant was interviewed with the Schedule for Affective Disorders and Schizophrenia for School Age Children-Present and Lifetime Version (Kiddie-SADS-PL) at the CAP units to confirm the ADHD symptoms and assess psychiatric comorbidity. The ADHD symptoms were confirmed by a parent rater within the last 3 years of inclusion for 94% of the population. Ninety-one percent of the participants were receiving pharmacological treatment for ADHD. Ninety-two percent had previously received a short psychoeducational intervention of 1–5 h, which typically consisted of information about ADHD diagnosis, symptoms, causes, and treatment options delivered by the patient‘s clinician to the patient with and/or without parents. Parents and a schoolteacher of each participant were also offered a standardized full-day lecture, with information about ADHD, pharmacotherapy, psychosocial interventions, and school interventions. Seventy-two percent of the participants attended this lecture. All the participants had previously had a collaborative meeting with the patient‘s parents and a schoolteacher to discuss supportive measures in school. These interventions were all completed before the baseline collection of data used in the present study.

**Table 1:** Characteristics of the participants (*n* = 100).

**Characteristic**	**M (SD)**
Age, years	15.8 (1.3)
Full-scale IQ (*n* = 86)	93.9 (13.0)
**Characteristic**	***n*** = **%**
Male patients	43
**ADHD presentation (Kiddies-SADS-PL)**	
ADHD-Predominantly inattentive	35
ADHD-Predominantly combined	31
Subtreshold ADHD	34
**Medication**	
^a^ADHD medication	91
^b^ Other pharmacological treatment	7
^c^ **Psychiatric comorbidities**	
Anxiety disorders	37
Posttraumatic stress disorder	1
Depressive disorder NOS/ Dysthymic disorder	11
Obsessive compulsive disorder	3
Tics disorder or Tourette‘s disorder	9
ODD/ Disruptive behavior disorder NOS	11
Learning Disorders, reading disorders or mixed	18

M mean, SD, standard deviation; Full scale IQ Wechsler Intelligence Scale for Children or Adults (WISC-IV, WAIS-IV), ADHD attention deficit/hyperactivity disorder,

a ADHD medication includes methylphenidate, lisdexamfetamine, atomoxetine, and guanfacine.

b Other pharmacological treatment includes neuroleptic medication; risperidone, quetiapine; anti-epileptic medication: valproate, lamotrigine.

c Psychiatric comorbidities are based on Kiddie-SADS-PL interview with the adolescents and converted to DSM-5 diagnoses. ODD, Oppositional Defiant Disorder; NOS, Not Otherwise Specified.

The socioeconomic status (SES) of the participants refers to the highest level of education reported by one or both parents (*n* = 75, 75%). This information was retrieved from the participants‘ medical records. Nineteen percent reported that they had an education lower than elementary school or 1–2 years of high school (0–11 years), seven percent completed high school and/or 1 year of training after high school (12–13 years), thirty percent had an academy or university degree for up to 4 years (14–15 years), and nineteen percent confirmed an academy/ university degree for 4 years or more (16 years and more).

### Measures

#### ADHD symptoms

The ADHD Rating Scale IV (ADHD RS-IV) (42) was used to measure the ADHD symptoms of the participants. We used the parent and self-report versions in this study. The ADHD RS-IV is an 18-item scale that assesses nine symptoms of inattention and nine symptoms of hyperactivity. The items correspond to the DSM-5 ADHD diagnostic criteria, including the combined and predominantly inattentive and hyperactive presentations. Item responses are scored from 0 = not at all to 3 = very often, with higher scores indicating more symptoms. The questionnaire has been validated for patients with ADHD aged 6–18 years across several European countries, including Norway, with impressive evidence for cross-factorial cultural validity, internal consistency, and convergent and divergent validity (43). Cronbach's α = 0.78 to 0.81 on the ADHD RS-IV parent version and 0.80 to 0.84 on the self-report version were reported in the present study.

#### Anxiety symptoms

The Screen for Child Anxiety-Related Emotional Disorders (SCARED) (44) was used to measure anxiety symptoms. The SCARED is a 41-item self-report screening instrument that measures anxiety symptoms in children and adolescents aged 8 to 18 years. In addition to a total scale score, the instrument contains five subscales representing diagnostic symptoms of panic disorder, generalized anxiety, separation anxiety, social phobia, and school phobia. Item responses are scored from 0 = not at all to 2 = often, and a total score ≥ 25 may indicate the presence of an anxiety disorder (45). The SCARED has shown good internal consistency and moderate parent-child correlations. The instrument is sensitive to detecting specific and/or comorbid anxiety diagnoses in children and adolescents (45). A study among Norwegian high school students found acceptable internal consistency (Cronbach‘s α = 0.62 to 0.87) for the SCARED subscales (46). Cronbach‘s α = 0.95 for the SCARED total score was reported in the current study.

#### Executive functions

The Behavior Rating Inventory of Executive Function (BRIEF) (22) assesses EF behaviors in children and adolescents at home and at school. The BRIEF includes an 86-item parent version (BRIEF-P) for children and adolescents aged 6–18 years and an 80-item self-report (BRIEF-SR) for children and adolescents aged 11–18 years (47). Both questionnaires contain a metacognitive index (MI), a behavior regulation index (BRI) and a global executive composite (GEC) score that represents the total scale score. The BRIEF-P and BRIEF-SR contain the following MI subscales: working memory, planning/organizing, organization of material and task completion. The BRI includes the following subscales: inhibit, shift and emotional control. Item responses are scored from 0 = not true to 2 = very true, with higher scores representing more severe dysfunction. According to the BRIEF manual, a total T-score above 65 indicates executive dysfunction. In this study, we used the MI, BRI and GEC scores. The BRIEF-P has shown good psychometric properties in a Norwegian child and adolescent population (48). Fallmyr and Egeland (48) found the Norwegian and American norms to be compatible, the questionnaire showed a good ability to discriminate between a normative population and a clinical ADHD population, and the internal consistency was acceptable (Cronbach's α = 0.76 to 0.92). The BRIEF-SR has shown acceptable psychometric properties in an American adolescent population, with α = 0.96 for the GEC and α = 0.72 to 0.96 for the clinical scales. Interrater reliability between the GEC of the BRIEF-P and the GEC of the BRIEF-SR was strong (r = 0.56) (47).

#### Functional impairment

The Weiss Functional Impairment Rating Scale parent-report (WFIRS-P) and self-report (WFIRS-S) versions (49) assess functional impairment in different domains typically affected in ADHD. The WFIRS-P and WFIRS-S consist of 50 and 69 items, respectively, divided into six and seven domains of impairment. The domains include family, school and learning, life skills, self-concept, social activities, and risky activities. The WFIRS-P and WFIRS-S are not parallel forms, but there are many parallel items. Item responses are scored from 0 = not at all to 3 = very often and 4 = not applicable, with higher scores indicating more impairment. In this study, a total mean score was calculated, representing the sum of the mean domain scores. Items with a missing or “not applicable” response were omitted. Any domain with a mean score > 1.5, two items with a score ≥ 2, or one item with a score = 3 is considered clinically impaired. The WFIRS scales have shown acceptable psychometric properties in a Norwegian adolescent ADHD population (50). In the present study, Cronbach's α was r = 0.62 to 0.88 for the WFIRS-P subscales and 0.70 to 0.92 for the WFIRS-S subscales.

### Statistical analyses

We conducted regression analyses to analyse the individual contributions of parent-rated and self-rated ADHD symptoms, executive dysfunction, and functional impairment to the severity of adolescent-rated anxiety symptoms in separate analyses. First, the ADHD RS-IV total score; the ADHD RS inattentive and the hyperactivity subscale scores; the BRIEF GEC, BRI and MI indices; and the WFIRS total score were entered one by one as independent variables, with the SCARED total score as the dependent variable. Second, to explore which of the subscales or indices from the ADHD RS-IV and the BRIEF questionnaire that predict anxiety the most while controlling for the other questionnaire subscale, we carried out two separate regression analyses: first with the two ADHD RS-IV subscales simultaneously and second with the two BRIEF indices simultaneously. Multicollinearity was checked to avoid high correlations (r =0.70 and above) between the independent variables, as recommended by Dormann et al. (51). The normality of residuals was checked by visual inspection of QQ plots.

**Figure 2:**
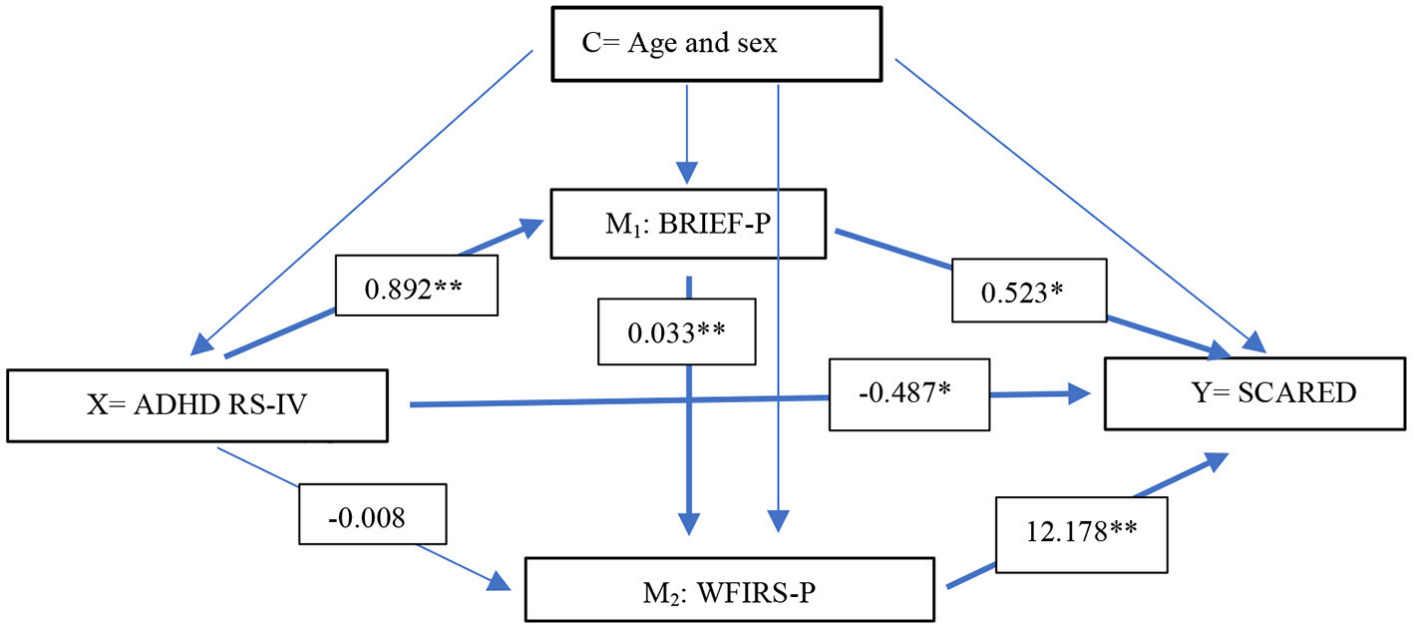
Regression coefficients for the associations between parent-report measures of ADHD RS-IV and SCARED (self-report) with BRIEF and WFIRS as possible mediators. Age and sex were considered confounders (*n* = 90). *X* predictor, *Y* dependent variable, *C* confounder, *M*_1_ mediator 1, *M*_2_ mediator 2. *ADHD RS-IV* Attention-Deficit/Hyperactivity Disorder Rating Scale*, BRIEF-P* Behavior Rating Inventory of Executive Function Parent version, *WFIRS* Weiss Functional Impairment Rating Scale. ** *p* < 0.005, * *p* < 0.05.

**Figure 3:**
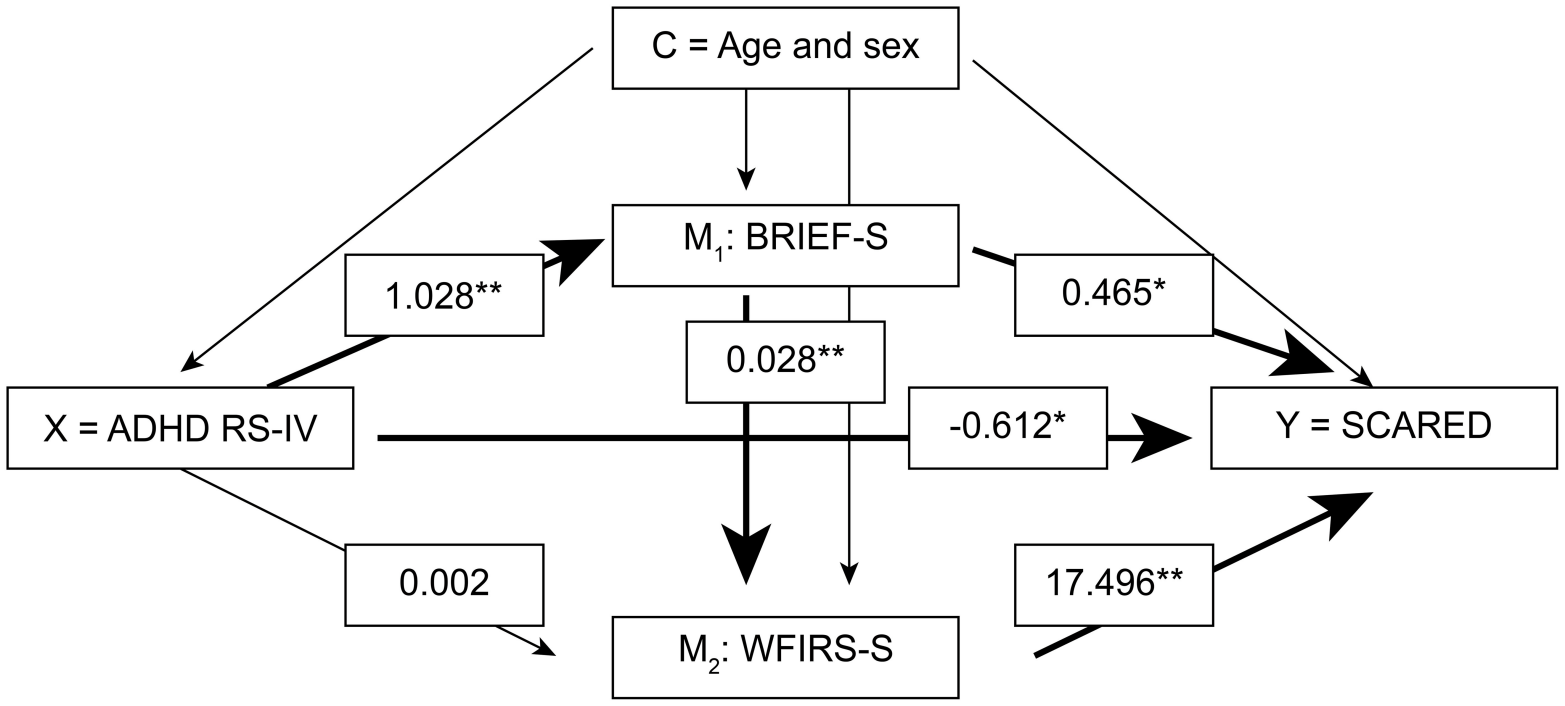
Regression coefficients for the associations between self-report measures of ADHD RS-IV and SCARED with BRIEF and WFIRS as possible mediators. Age and sex were considered confounders (*n* = 81). *X* predictor, *Y* dependent variable, *C* confounder, *M*_1_ mediator 1, *M*_2_ mediator 2, *ADHD RS-IV* Attention-Deficit/Hyperactivity Disorder Rating Scale*, BRIEF-S* Behavior Rating Inventory of Executive Function Self-report, *WFIRS* Weiss Functional Impairment Rating Scale Self-report. ** *p* < 0.005, * *p* < 0.05.

To explore whether EFs and functional impairment act as mediators in the association between ADHD symptoms and anxiety, we conducted a serial multiple mediator model recommended by Hayes (52) using the PROCESS macro for IBM SPSS (www.processmacro.org). The PROCESS command generates bootstrap confidence intervals (CIs) for all indirect effects as well as possible pairwise comparisons between indirect effects. We used 5,000 bootstrap samples. The ADHD RS total score was entered as the predictor variable (X), the BRIEF GEC score was entered as the first mediator (M^1^), and the WFIRS total score was entered as the second parallel mediator (M^2^) (see Figures 2, 3). The adolescent- rated SCARED total score was the outcome variable in both mediation analyses. The analyses were conducted using parent- and self-report questionnaires in separate analyses. Age and sex were considered confounders and entered as covariates in all the analyses. In addition, we carried out supplementary analyses also adjusting for IQ and SES. These analyses were restricted to the 62 participants in the regression analyses and 58 and 52 participants in the mediation analyses using parent- and self-reports, respectively, with available data on these variables. The estimated effects were essentially the same (data not shown). Missing data were handled using single imputation on scales using the mean score if 70% or more of the questions were answered. Otherwise, the outcome of that specific questionnaire for that participant was treated as missing. For the rest, we handled missing data using available case analyses, including in each analysis the cases with data on the variables in the analysis. We report 95% CIs where relevant. To reduce the risk of false-positive findings due to multiple hypotheses, two-sided *p*-values ≤ 0.01 are regarded as significant. This approach is suggested by Lydersen (53). Statistical analyses were conducted using SPSS 26.

## Results

### Clinical characteristics of the participants

The 100 participants included 57 females and 43 males; the mean age was 15.8 years (SD = 1.3). When collecting the data, ninety-one percent of the participants were stably taking ADHD medication. Nevertheless, sixty-six percent of participants met the DSM-5 criteria for an ADHD diagnosis (35% predominantly inattentive presentation and 31% combined presentation), and 33% presented subthreshold symptoms of ADHD (all ADHD medicated). Fifty-three percent of the participants had at least one current comorbid condition; among them, 37% had an AD according to the DSM-5 criteria (see Table 1).

### Results from the regression analyses

The results from the regression analyses using parent-ratings of the ADHD RS-IV, the BRIEF and the WFIRS to predict anxiety symptoms measured with the SCARED self-report, controlling for age and sex, are presented in Table 2. In step 1, neither the ADHD total score, nor the subscale scores were able to predict anxiety. All the BRIEF indices, including the GEC (*p* = 0.002), the BRI (*p* = 0.010) and the MI (*p* = 0.003) significantly predicted anxiety, as did the WFIRS total score (*p* < 0.001).

**Table 2:** Results from regression analysis using the parent-rated ADHD RS-IV, BRIEF and WFIRS measures as independent variables entered in two steps.

**Measures**	**Step 1**				**Step 2**			
	**Regression coefficient B**	**95% confidence interval**			**Regression coefficient B**	**95% confidence interval**		
		**Lower**	**Upper**	***p*-value**		**Lower**	**Upper**	***p*-value**
**ADHD RS-IV parent total**	0.181	−0.157	0.518	0.290				
Inattentive subscale	0.606	0.020	1.191	0.043	0.715	0.079	1.351	0.028
Hyperactivity subscale	−0.005	−0.539	0.528	0.984	−0.253	−0.822	0.316	0.379
**BRIEF parent GEC**	0.440	0.167	0.712	0.002^*^				
BRI	0.321	0.078	0.564	0.010^*^	0.153	−0.153	0.459	0.323
MI	0.429	0.147	0.711	0.003^*^	0.318	0.040	0.677	0.081

SCARED self-report is the dependent variable in all the analyses. All analyses were adjusted for age and sex (n = 92). Step 1 only one single independent variable is entered in the analyses, step 2 the subscales in the same questionnaire are entered simultaneously as independent variables. The Screen for Children Anxiety Related Emotional Disorders (SCARED) is the dependent variable in all the analyses. ADHD-RS-IV Attention- Deficit/Hyperactivity Disorder Rating Scale, BRIEF Behavior Rating Inventory of Executive Function, WFIRS Weiss Functional Impairment Rating Scale, GEC Global Executive Composite, BRI Behavior Regulation Index, MI Metacognitive Index. ^**^p < 0.001, ^*^p < 0.01.

In step 2 of Table 2, we examined the ability of the ADHD RS-IV subscale scores and the BRIEF indices to predict anxiety when controlling for age, sex, and the companion subscale or index score. The results showed that neither the ADHD subscales nor the BRIEF indices were able to predict anxiety when controlling for age, sex, and the companion subscale.

The results from the regression analyses using self-ratings of the ADHD RS-IV, the BRIEF and the WFIRS to predict anxiety symptoms measured with SCARED, controlling for age and sex, are presented in Table 3. In step 1, the ADHD inattentive subscale score was significantly able to predict anxiety (*p* = 0.002). Among the BRIEF indices, the GEC, MI, and BRI predicted anxiety (*p* ≤ 0.001), as did the total score of the WFIRS (*p* < 0.001).

**Table 3:** Results from regression analyses using self-report measures of the ADHD RS-IV, BRIEF and WFIRS measures entered as independent variables in two steps.

**Measures**	**Step 1**				**Step 2**			
	**Regression coefficient B**	**95% confidence interval**			**Regression coefficient B**	**95% confidence interval**		
		**Lower**	**Upper**	***p*-value**		**Lower**	**Upper**	***p*-value**
**ADHD RS-IV self total**	0.342	0.036	0.649	0.029				
Inattentive subscale	0.818	0.315	1.321	0.002^**^	0.985	0.380	1.590	0.002^*^
Hyperactivity subscale	0.211	−0.337	0.760	0.445	−0.318	−0.931	0.294	0.304
**BRIEF self GEC**	0.484	0.271	0.697	<0.001^**^				
BRI	0.371	0.166	0.575	0.001^**^	0.161	−0.098	0.419	0.220
MI	0.481	0.259	0.704	<0.001^**^	0.366	0.078	0.655	0.013
WFIRS self-report total	16.929	11.738	22.121	<0.001^**^				

SCARED self-report is the dependent variable in all the analyses. All analyses were adjusted for age and sex (n = 92). Step 1 only one single independent variable is entered in the analyses, step 2 the subscales in the same questionnaire are entered simultaneously as independent variables. The Screen for Children Anxiety Related Emotional Disorders (SCARED) is the dependent variable in all the analyses. ADHD-RS-IV Attention- Deficit/Hyperactivity Disorder Rating Scale, BRIEF Behavior Rating Inventory of Executive Function, WFIRS Weiss Functional Impairment Rating Scale, GEC Global Executive Composite, BRI, Behavior Regulation Index; MI, Metacognitive Index.

** p < 0.001,

* p < 0.01.

In step 2 of Table 3, we examined the ability of the ADHD RS-IV subscale scores and the BRIEF indices to predict anxiety when controlling for age, sex, and the companion subscale or index score. The results showed that the ADHD inattention score was able to predict anxiety when controlling for the hyperactive subscale score. The BRIEF indices were unable to predict anxiety when controlling for each other.

### The role of EFs and functional impairment in mediating the association between ADHD symptoms and anxiety

Regression coefficients presenting the direct effects between the parent-rated variables are presented in Figure 2. The total direct and indirect effects between the variables are presented in Supplementary Table S4. The direct effect of ADHD symptoms predicting anxiety was negative but significant (coefficient = −0.487, *p* = 0.048); thus, a low ADHD symptom score significantly predicted more severe symptoms of anxiety when EFs and functional impairment were controlled for. The first indirect effect of only EF mediating the association between ADHD symptoms and anxiety was significantly positive (coefficient = 0.466, CI 0.013 to 1.024). Thus, levels of ADHD symptoms predicted levels of executive dysfunction, which again mediated the severity of anxiety symptoms. The second indirect effect was through levels of functional impairment only, which was negative and non-significant (coefficient = −0.101, CI:−0.275 to 0.0241). The third indirect effect of executive dysfunction and functional impairment in serial (X- M^1^- M^2^-Y) was positive and significant (coefficient = 0.362, CI: 0.087 to 0.665); thus, the association between ADHD symptoms and anxiety seems to be mediated by EFs affecting functional impairment, which in turn mediates symptoms of anxiety. This was found regardless of age, sex, IQ, and SES.

Regression coefficients presenting the direct effects between the self-rated variables are presented in Figure 3. The total direct and indirect effects between the variables are presented in Supplementary Table S5. The results from the self-report measures were similar to the results from the parent reports, showing a negative but significant direct effect of ADHD symptoms predicting anxiety (coefficient = −0.612, *p* = 0.007). The first indirect effect *via* executive dysfunction alone was positive and significant (coefficient = 0.478, CI: 0.025 to 0.947), while the second indirect effect *via* functional impairment alone was non-significant (coefficient = 0.035, CI:−0.199 to 0.261). The serial indirect effect of executive dysfunction and functional impairment on the association between ADHD and anxiety was positive and significant (coefficient = 0.501, CI: 0.203 to 0.889), and the results were similar to the analyses with the parent reports.

## Discussion

Children and adolescents with ADHD have an increased risk of comorbid ADs compared to their non-ADHD peers. In addition, anxiety symptoms tend to have an earlier onset, be more severe and be frequently associated with other psychiatric conditions (54, 55). Although the comorbidity rates of ADHD and ADs are well documented, the etiology of these associations is poorly understood. As such, the first aim of the current study was to examine whether parent- and self-rated ADHD symptoms, executive dysfunction and functional impairment were able to predict anxiety in a clinical population of adolescents with symptoms of ADHD. Overall, the main patterns were similar in the adolescent and parent reports, with stronger associations in the self-reports. This finding was as expected, considering that anxiety is an internalizing disorder and that only the adolescents reported anxiety symptoms. Among the self-rated ADHD symptom scores, only the inattention domain predicted anxiety, while hyperactivity symptoms were unable to predict anxiety. This was found regardless of age and sex and is in line with previous studies showing inattention symptoms to be more strongly associated with anxiety than hyperactivity (14, 56). On the other hand, none of the parent-rated ADHD symptom scores were able to predict anxiety. This was somewhat surprising and might suggest that parents are less sensitive in capturing this symptom association, perhaps because of the less overt nature of both inattention symptoms and anxiety symptoms. On the other hand, only including a self-report measure of anxiety could have affected this finding.

Among the EFs, both the global executive composite (GEC), the behavioral regulation index (BRI) and the metacognitive index (MI) were able to predict anxiety in both self- and parent-reports. Neither the BRI nor the MI index was significantly able to predict anxiety over and above the other BRIEF index when controlling for age and sex. The behavioral regulation index reflects both emotional dysregulation, mental inflexibility and impulsivity. An association between emotional dysregulation and anxiety would be expected. In addition, an association between mental inflexibility and anxiousness has previously been found in studies examining measures of shifting and updating using neurocognitive measures on clinical and non-clinical samples of anxious children and adolescents (57–59). Impulsivity, on the other hand, has previously been shown to be inversely associated with anxiety, with studies showing less inhibited children and adolescents presenting lower symptoms of anxiety than more inhibited children (60, 61). More detailed subgroup analyses are recommended to explore these associations further.

The metacognitive index includes cognitive functions such as working memory, task completion, planning and organization, and organization of materials, all skills strongly related to school performance and the attainment of future goals. As such, it is reasonable to link experienced difficulties in these areas with both low self-esteem and lack of control, which again may increase anxiousness. Finally, the functional impairment score, representing self-concept, social activities, school functioning, life skills and risky activities, predicted anxiety in both the parent- and self-reports. In sum, both executive dysfunction related to behavior regulation and metacognitive functions and functional impairment may be important to assess when working with adolescents with ADHD and comorbid anxiety.

The second aim of this study was to explore the roles of executive dysfunction and functional impairment as possible mediators in the ADHD-anxiety relationship. The results from the mediation analyses showed that ADHD symptoms alone were unable to predict anxiety when controlling for executive dysfunction and functional impairment. Executive dysfunction mediated the ADHD-anxiety pathway, as expected; thus, more ADHD symptoms significantly predicted more severe executive dysfunction, which mediated the severity of anxiety symptoms. This pattern was found when controlling for age and sex and was similar in both parent- and self-reports, strengthening the validity of the result. This result was similar to a previous study (37) where EFs and functional impairment explained most of the variance in combined anxiety and depressive symptoms in college students with ADHD. Another recent study found that EFs and anxiety/depression significantly mediate the relationship between ADHD and quality of life (62). Together, these results suggest that executive dysfunctions rather than ADHD symptoms *per se* may lead to comorbid anxiety or mood disorders and difficulties in daily life. In the current study, ADHD symptoms alone were unable to predict functional impairment, but functional impairment predicted anxiety. Functional impairment was thus unable to mediate the ADHD-anxiety relationship by itself but acted as a mediator through EFs. Executive dysfunction has previously been linked to functional impairment in both school and social settings in children and adolescents with ADHD (63, 64). Rosellȯ et al. (65) found impulsivity/emotional lability as well as planning and organizing to be significant predictors of functional impairment in family, social, academic, and risky activity areas in young adults with ADHD. Shift and working memory marginally predicted impairment in the same areas. These findings suggest executive dysfunction to be an important target in the prevention of functional impairment across several life domains.

This study has several strengths. First, this was the first study to explore the mediating effect of EFs and functional impairment on the ADHD-anxiety relationship in a clinical adolescent population. Second, we included ADHD patients with common psychiatric comorbidities, which increases the generalizability of the results to a clinical CAP setting. Third, the use of both parent- and self-report measures improves the validity of the results. Finally, males and females were equally represented among the participants, which is positive when controlling for the possible moderating effect of sex on the ADHD-anxiety relationship. This study also has several limitations. First, most of the participants (91.0%) were on ADHD medication when completing the questionnaires; thus, the results may not be representative of adolescents with ADHD who are not on medication. Second, the participants showed subthreshold ADHD symptoms, which may limit the generalizability to participants with more severe symptoms. Third, data on ADHD symptoms, executive functions and functional impairments were restricted to parent- and self-report questionnaires. The additional use of neurocognitive measures and computer tests to assess inattention symptoms and executive functions could have added valuable supplemental information in this study. Fourth, a high correlation between the main index scores (see Supplementary Table S6) indicates some conceptual overlap between the EF and functional impairment questionnaires. The two measures, however, also represent distinct, non-overlapping problem areas, making each questionnaire valuable as a clinical assessment instrument. Fifth, since this is a cross-sectional observational study, we have no longitudinal data to explore different developmental pathways related to the included variables. Moreover, the inclusion of only quantitative data limits an in-depth understanding of the origin and consequence of the different associations between cognitive functions/ADHD symptoms, functional impairments, and anxiety in this adolescent population. The inclusion of qualitative methods, such as interviewing parents and adolescents about life conditions, symptom development, psychosocial treatment and medication effects, could improve our knowledge of these associations for different patient groups and is recommended for use in future studies.

## Conclusion and relevance

In this study, ADHD inattention symptoms, executive functions and functional impairment predicted anxiety in a clinical adolescent population with ADHD symptoms. ADHD core symptoms were, however, not able to predict anxiety when controlling for executive functions and functional impairment in a mediation analysis. Executive dysfunction acted as a substantial mediator in this relationship, while functional impairment mediated this relationship only through EFs. This result pinpoints executive dysfunction as an important treatment target in alleviating anxiety in adolescents with impairing ADHD symptoms. Thus, the additional assessment of EFs using a broadband questionnaire such as the BRIEF parent- and self-report versions is recommended when assessing ADHD and comorbid disorders in adolescent populations. The results also suggest that adolescents with ADHD with normal EFs may have a lower risk of experiencing anxiety. These results improve our knowledge of the association between ADHD and anxiety in a clinical ADHD population. More studies are needed to replicate these findings across different age groups.

## Data availability statement

The original contributions presented in the study are included in the article/Supplementary material, further inquiries can be directed to the corresponding author.

## Ethics statement

The studies involving human participants were reviewed and approved by the Regional Committee for Medical and Health Research Ethics in Southeast Norway (2015/2115). Written informed consent to participate in this study was provided by the participants' legal guardian/next of kin.

## Author contributions

TN, PT, and SL supervised A-LH. A-LH drafted the paper and performed the statistical analyses with the supervision of SL. All authors contributed to the research design, provided substantial contributions to the paper, and read and approved the final version of the manuscript.

## Funding

The study was funded by a PhD grant to A-LH by the Regional Centre for Child and Youth Mental Health and Child Welfare (RKBU), Faculty of Medicine and Health Sciences, Norwegian University of Science and Technology (NTNU). The study received additional funding from the Child and Adolescent Psychiatric Clinic, St. Olav's University Hospital; the Regional Network for Autism, ADHD, and Tourette syndrome, Mid-Norway Health Trust; the National Research Network for ADHD, Ullevål University Hospital, Oslo; and the Regional Competence Network for ADHD, RKBU, NTNU.

## Conflict of interest

Authors A-LH, AS, and TN have received a speaker's fee and a travel honorarium from Medice during the last 3 years. Author PT has received speakers fee from Medice and Takeda within last 3 years. Author PT has received royalties from several publishers for books on ADHD and related disorders. The remaining author declares that the research was conducted in the absence of any commercial or financial relationships that could be construed as a potential conflict of interest.

## Publisher's note

All claims expressed in this article are solely those of the authors and do not necessarily represent those of their affiliated organizations, or those of the publisher, the editors and the reviewers. Any product that may be evaluated in this article, or claim that may be made by its manufacturer, is not guaranteed or endorsed by the publisher.

## Abbreviations

ADHD: Attention-deficit/hyperactivity disorder
ADHD-RS IV: ADHD Rating Scale IV
BRIEF: The Behavior Rating Inventory of Executive Function
CAP: Child and Adolescent Psychiatry
CI: Confidence Intervals
EF: Executive Functions
GAD: Generalized Anxiety Disorder
SCARED: The Screen for Child Anxiety-Related Emotional Disorders
WFIRS-P/-S: Weiss Functional Impairment Rating Scale parent-report/self-report versions.
