# Supplementary material for: Executive functions mediate the association between ADHD symptoms and anxiety in a clinical adolescent population
Source: Front Psychiatry. 2022 Sep 12;13:834356. doi: 10.3389/fpsyt.2022.834356 (PMC9510657; doi:10.3389/fpsyt.2022.834356)
Supplement: Supplementary file 1 [file Table_1.DOCX]

**Supplemental Tables**

Supplemental Table 1. Scale scores on measures of ADHD RS-IV, BRIEF, WFIRS and SCARED.

| Measures Scores  min-max Mean SD N | | | | |
| --- | --- | --- | --- | --- |
| ADHD RS- IV Parent Total | 4 -84 | 25.0 | 8.8 | 97 |
| Inattentive symptoms | 3-27 | 15.5 | 5.1 | 98 |
| Hyperactive/Impulsive symptoms | 0-21 | 9.4 | 5.5 | 99 |
| ADHD-RS IV Self Total | 1-41 | 21.5 | 9.9 | 91 |
| Inattentive symptoms | 0-23 | 11.8 | 5.7 | 96 |
| Hyperactive/Impulsive symptoms | 0-23 | 9.7 | 5.6 | 93 |
| BRIEF-Parent General Executive Composite (GEC) T-score | 41-91 | 68.0 | 10.4 | 100 |
| BRIEF Parent Behavior Regulation Index (BRI) T-score | 39-94 | 63.6 | 11.9 | 100 |
| BRIEF Parent Metacognitive index (MI) T-score | 41-90 | 68.9 | 10.2 | 100 |
| Inhibit | 41-94 | 61.0 | 13.8 | 100 |
| Shift | 38-98 | 63.0 | 11.5 | 100 |
| Emotional Control | 40-87 | 62.0 | 11.7 | 100 |
| Initiate | 36-86 | 63.7 | 10.8 | 100 |
| Working Memory | 46-92 | 72.7 | 10.4 | 100 |
| Plan Organize | 43-91 | 69.6 | 10.3 | 100 |
| Organization of Materials | 34-72 | 57.4 | 10.4 | 100 |
| Monitor | 36-91 | 63.6 | 11.4 | 100 |
| BRIEF-Self General Executive Composite (GEC) | 30-94 | 63.9 | 13.1 | 100 |
| BRIEF Self Behavior Regulation Index (BRI) | 31-91 | 59.7 | 14.0 | 100 |
| BRIEF Self Metacognitive index (MI) | 31-91 | 65.6 | 12.7 | 100 |
| Inhibit | 34-93 | 59.1 | 14.0 | 100 |
| Shift | 32-83 | 58.5 | 13.1 | 100 |
| Emotional Control | 34-84 | 57.1 | 13.5 | 100 |
| Monitor | 36-83 | 55.3 | 12.2 | 100 |
| Working Memory | 34-87 | 66.3 | 12.1 | 100 |
| Plan/ Organize | 31-86 | 61.0 | 12.3 | 100 |
| Organization of Materials | 33-84 | 58.0 | 12.3 | 100 |
| Task Completion | 35-88 | 66.8 | 12.4 | 100 |
| WFIRS-Parent total mean score | 0.0-2.6 | 0.8 | 0.4 | 96 |
| Family | 0.0-2.6 | 0.8 | 0.6 | 99 |
| School and Learning | 0.0-3.0 | 0.9 | 0.6 | 98 |
| Life skills | 0.0-3.0 | 1.1 | 0.5 | 100 |
| Self esteem | 0.0-3.0 | 1.1 | 0.8 | 99 |
| Social | 0.0-5.7 | 0.7 | 0.8 | 100 |
| Risky Activitites | 0.0-2.0 | 0.2 | 0.3 | 99 |
| WFIRS-Self total mean score | 0.0-2.0 | 0.8 | 0.5 | 98 |
| Family | 0.0-2.3 | 0.8 | 0.6 | 96 |
| School and Learning | 0.0-2.5 | 1.0 | 0.6 | 98 |
| Life skills | 0.0-2.6 | 1.0 | 0.6 | 99 |
| Self esteem | 0.0-3.0 | 1.1 | 0.9 | 98 |
| Social | 0.0-2.3 | 0.6 | 0.5 | 99 |
| Risky Activities | 0.0-2.5 | 0.3 | 0.4 | 89 |
| Screen for Child Anxiety Related Emotional Disorders Total | 0-64 | 21.9 | 15.5 | 92 |
| Note: *ADHD RS-IV* Attention Deficit/Hyperactivity Disorder Rating Scale IV*, WFIRS* Weiss Functional Impairment Rating Scale*, BRIEF* Behavior Rating Inventory of Executive Function | | | |  |

Supplemental Table 2. Regression coefficients, Standard Errors and model summary information for the mediator model using the parent-report of ADHD RS-IV, BRIEF, WFIRS and SCARED (n=90).

| Consequent | | | | | | | | | |
| --- | --- | --- | --- | --- | --- | --- | --- | --- | --- |
|  | M_1_ (BRIEF) | | | M_2_ (WFIRS) | | | Y (SCARED) | | |
| Antecedent | Coeff. | SE | p | Coeff. | SE | p | Coeff. | SE | p |
| X (ADHD RS-IV) | 0.892 | 0.077 | <.001 | -0.008 | 0.006 | .186 | -0.487 | 0.243 | .048 |
| M_1_ BRIEF (GEC) | -- | -- | -- | 0.033 | 0.005 | <.001 | 0.523 | 0.253 | .041 |
| M_2_ WFIRS total | -- | -- | -- | -- | -- | -- | 12.178 | 4.186 | .005 |
| SEX | 0.301 | 1.364 | .826 | -0.001 | 0.069 | .991 | 13.628 | 2.664 | <.001 |
| AGE | -0.583 | 0.518 | .264 | < 0.001 | 0.026 | .996 | -0.206 | 1.019 | .840 |
| Constant | 54.185 | 8.683 | <.001 | -1.235 | .529 | .022 | -29.718 | 21.073 | .162 |
|  | | | | | | | | | |
| Note: *ADHD RS-IV* Attention Deficit Hyperactivity Disorder Rating Scale IV, *BRIEF* Behaviour Rating Inventory of Executive Functions, *WFIRS* The Weiss Functional Impairment Rating Scale*, SCARED* Screen for Child Anxiety-Related Emotional Disorders. Sex and age were confounders, *M* Mediator. | | | | | | | | | |

Supplemental Table 3. Regression coefficients, Standard Errors and model summary information for the mediator model using self-report of the ADHD RS-IV, BRIEF, WFIRS and SCARED (n=81).

| Consequent | | | | | | | | | |
| --- | --- | --- | --- | --- | --- | --- | --- | --- | --- |
|  | M_1_ (BRIEF) | | | M_2_ (WFIRS) | | | Y (SCARED) | | |
| Antecedent | Coeff. | SE | p | Coeff. | SE | p | Coeff. | SE | p |
| X (ADHD RS-IV) | 1.028 | 0.081 | <.001 | 0.002 | 0.006 | .753 | -0.612 | 0.22 | .007 |
| M_1_ BRIEF (GEC) | -- | -- | -- | 0.028 | 0.005 | <.001 | 0.465 | 0.207 | .028 |
| M_2_ WFIRS total | -- | -- | -- | -- | -- | -- | 17.496 | 3.937 | <.001 |
| SEX | 5.124 | 1.655 | .003 | -0.184 | 0.079 | 0.023 | 11.019 | 2.805 | <.001 |
| AGE | 0.018 | 0.629 | .978 | -0.011 | 0.028 | 0.695 | -1.191 | 0.973 | .225 |
| Constant | 33.806 | 9.784 | <.001 | -0.496 | .473 | .298 | -8.069 | 16.352 | .623 |
| Note: *ADHD RS-IV* Attention Deficit Hyperactivity Disorder Rating Scale IV, *BRIEF* Behaviour Rating Inventory of Executive Functions, *WFIRS* The Weiss Functional Impairment Rating Scale*, SCARED* Screen for Child Anxiety-Related Emotional Disorders. Sex and age were confounders, *M* Mediator. | | | | | | | | | |

Supplemental Table 4. Regression coefficients with total-, and indirect effects of the association between parent-reports of ADHD RS-IV (X) and SCARED (Y), with BRIEF and WFIRS as mediators (M) (n=90).

| Associations between variables | Coefficient/  effect | Standard  error | CI | p |
| --- | --- | --- | --- | --- |
| Total effect (direct and indirect effects) of X on Y | 0.240 | 0.171 | -0.101 to 0.581 | 0.165 |
|  | | | | |
| Indirect effect of X on Y | Coefficient/  effect | Bootstrap  Standard error | CI |  |
| Total indirect effect (a+b+c) | 0.727* | 0.251 | 0.283 to 1.268 |  |
| ^a^ADHD RS-BRIEF-SCARED | 0.466* | 0.261 | 0.013 to 1.024 |  |
| ^b^ADHD RS-WFIRS-SCARED | -0.101 | 0.078 | -0.275 to 0.024 |  |
| ^c^ADHD RS-BRIEF-WFIRS-SCARED | 0.362* | 0.150 | 0.087 to 0.665 |  |
| Note Results from PROCESS procedure for SPSS (Hayes, 2018.www.guilford.com/p/hayes3). The total effect = the sum of direct and indirevt effets ^a^ The first indirect effect, ^b^ The second indirect effect, ^c^ The third indirect effect *CI* Confidence Intervals (95%). | | | | |

Supplemental Table 5. Regression coefficients with total-, and indirect effects of the association between self-reports of ADHD RS-IV (X) and SCARED (Y), with BRIEF and WFIRS as mediators (M) (n=81).

| Associations between variables | Coefficient/  effect | Standard  error | CI | p |
| --- | --- | --- | --- | --- |
| Total effect of X on Y | 0.402 | 0.159 | 0.087 to 0.718 | 0.132 |
|  | | | | |
| Indirect effect of X on Y | Coefficient/  effect | Bootstrap  Standard error | CI |  |
| Total indirect effect (a+b+c) | 1.015* | 0.220 | 0.598 to 1.471 |  |
| ^a^ADHD RS-BRIEF-SCARED | 0.478* | 0.234 | 0.025 to 0.947 |  |
| ^b^ADHD RS-WFIRS-SCARED | 0.035 | 0.115 | -0.200 to 0.261 |  |
| ^c^ADHD RS-BRIEF-WFIRS-SCARED | 0.501* | 0.177 | 0.203 to 0.889 |  |
|  | | | | |
| Note Results from PROCESS procedure for SPSS (Hayes, 2018.www.guilford.com/p/hayes3), ^a^ The first indirect effect, ^b^ The second indirect effect, ^c^ The third indirect effect. *CI* Confidence Intervals (95%) with 5000 bootstrap samples. | | | | |

Supplemental Table 6. Correlation matrix between the WFIRS total score and BRIEF GEC, MI and BRI indexes using parent- and self-reports.

| Measures | WFIRS-P  total score (n) | Measures | WFIRS-S  total score (n) |
| --- | --- | --- | --- |
| BRIEF-P GEC | 0.648** (99) | BRIEF-S GEC | 0.707** (98) |
| BRIEF-P BRI | 0.580** (99) | BRIEF-S BRI | 0.645** (98) |
| BRIEF-P MI | 0.585** (99) | BRIEF-S MI | 0.653** (98) |
| Note *WFIRS* Weiss Functional Impairment Rating Scale*, BRIEF* Behavior Rating Inventory of Executive Function, *GEC* General executive composite, *BRI* Behaviour regulation index, *MI* Metacognitive Index. **. Pearson correlation is significant at 0.01 level (2-tailed) | | | |
